# Load-Bearing Capacity of Zirconia Crowns Screwed to Multi-Unit Abutments with and without a Titanium Base: An In Vitro Pilot Study

**DOI:** 10.3390/ma12193056

**Published:** 2019-09-20

**Authors:** Hadas Heller, Adi Arieli, Ilan Beitlitum, Raphael Pilo, Shifra Levartovsky

**Affiliations:** 1 Department of Oral Rehabilitation, the Maurice and Gabriela Goldschleger School of Dental Medicine, Tel Aviv University, Tel Aviv, Israel; heller.hadas@gmail.com (H.H.); dr.arieli@gmail.com (A.A.);; 2 Department of Periodontology and Dental Implantology, Maurice and Gabriela Goldschleger School of Dental Medicine, Tel Aviv University, Tel Aviv, Israel; beilan1@bezeqint.net

**Keywords:** monolithic zirconia, multi-unit abutment, titanium base

## Abstract

The static and dynamic load-bearing capacities and failure modes of zirconia crowns screwed to multi-unit abutments (MUAs) with and without a titanium base (T-base) were determined. Thirty-six monolithic zirconia crowns screwed to straight MUAs torqued to laboratory analogs (30 Ncm) were assigned to two groups (n = 18). In group A, the zirconia crowns were screwed directly to the MUAs; in group B, the zirconia crowns were cemented to the T-base and screwed to the MUAs. All specimens were aged in 100% humidity (37 °C) for one month and subjected to thermocycling (20,000 cycles). Afterwards, the specimens underwent static and dynamic loading tests following ISO 14801. The failure modes were evaluated by stereomicroscopy (20×). There was an unequivocally similar trend in the S-N plots of both specimen groups. The load at which the specimens survived 5,000,000 cycles was 250 N for both groups. Group A failed mainly within the metal, and zirconia failure occurred only at a high loading force. Group B exhibited failure within the metal mostly in conjunction with adhesive failure between the zirconia and T-base. Zirconia restoration screwed directly to an MUA is a viable option, but further studies with larger sample sizes are warranted.

## 1. Introduction

As esthetic demands in dentistry increase, porcelain fused to metal has become more frequently substituted with zirconia-based ceramic for teeth and implant-supported fixed dental prostheses (FDPs) [1]. With the introduction of computer-aided design/computer-aided manufacturing (CAD/CAM) technologies and the development of yttrium oxide partially stabilized tetragonal zirconia polycrystalline (Y-TZP), bilaminar or preferably monolithic zirconia structures have become the material of choice. Monolithic zirconia restorations demonstrate a high flexural strength (900–1200 MPa) and fracture toughness (9–10 Mpa m^0.5^) compared to those of other ceramic materials and exhibit minimal wear of the antagonist teeth [2,3].

Implant-supported prostheses are utilized via two methods of retention; they are either screw-retained or cement-retained. Both modalities have benefits and shortcomings in clinical application [4]. In recent years, with the increased risk of implant loss due to biological complications such as peri-implantitis, which is associated mainly with cemented implant restorations, the advantages of retrievability as well as accessibility for maintenance and replacement have accelerated the use of screw-retained implant restorations. Due to these reasons and their apparently higher biological compatibility, the screw-retained modalities are currently preferable [5,6,7,8]. When connecting several implants with a screw-retained implant restoration, there is a need for an interim part, a multi-unit abutment (MUA), to correct the differences in implant angles and to create a common path of insertion. The first MUA was introduced for the Branemark implant system and was configured as a two-piece titanium abutment cylinder [9]. Currently, a one-piece abutment, which can be straight or angled, is commonly used. These definitive MUAs enable better hemidesmosomal adherence between the soft tissue and titanium and therefore might reduce bone resorption around the implants [10,11,12]. This “one abutment at one time” concept is especially advantageous in immediately restored implants for partial and full edentulous cases, whereas non removal of the multi-unit abutments placed at the time of surgery results in a statistically significant reduction in crestal bone resorption around the implants [11].

The high demand for aesthetic outcomes, combined with new material technologies and the need for retrievability in implant-supported FDPs, has led to an increased use of screwed monolithic zirconia implant-supported restorations. Usually, this type of zirconia is cemented to a milled titanium sleeve (T-base) and then screwed to the MUA (screwed-cemented type of restoration). This design was suggested by McGlumphy et al. [13], Rajan and Gunaseelan [14], and Uludag and Celik [15] and is known as “the combination implant crown”. Hussien et al. [16] showed that this type of combination (screwed-cemented) does not affect the fatigue failure load of monolithic zirconia, monolithic lithium disilicate, or veneered zirconia ceramic crowns when compared to cemented crowns. Moreover, significantly higher fatigue failure loads have been recorded for monolithic zirconia crowns than for the other two types of crowns [16]. The retention of monolithic zirconia copings to the T-base might be the weakest link of this type of screwed-cemented restoration; therefore, a reliable bond between the zirconia, cement, and T-base is essential for the longevity of the restoration [17,18].

In contrast to the screwed-cemented restoration, there is another mode of retention of monolithic zirconia crowns to MUAs: Screwing the zirconia directly to the MUA (screwed restoration) without the use of a T-base, thus avoiding the dependence on the cemented joint between the zirconia and the titanium. Although restorations with zirconia crowns attached to MUAs with and without titanium bases are widely used by clinicians, there is no evidence-based data to favor either of these approaches; moreover, no research study has compared the mechanical failure between the two methods. Some believe that the titanium base is vital for the survival of the zirconia restoration, while others are concerned with the detrimental impact of the cement that is placed between the zirconia and the titanium base.

The aim of the current study was to compare the static and dynamic load-bearing capacities and determine the failure mode of 3Y-TZP (zirconia) crowns screwed to MUAs with and without a T-base. The null hypothesis was that there would be no difference in either the failure load or the failure mode between the two groups.

## 2. Results

Table 1 presents the results of the static load compressive test for three specimens of each group. From the mean maximal failure load, the 80% level load was set for the start of the dynamic test. The 80% level set for group A was slightly higher than that for group B.

**Figure 1:**
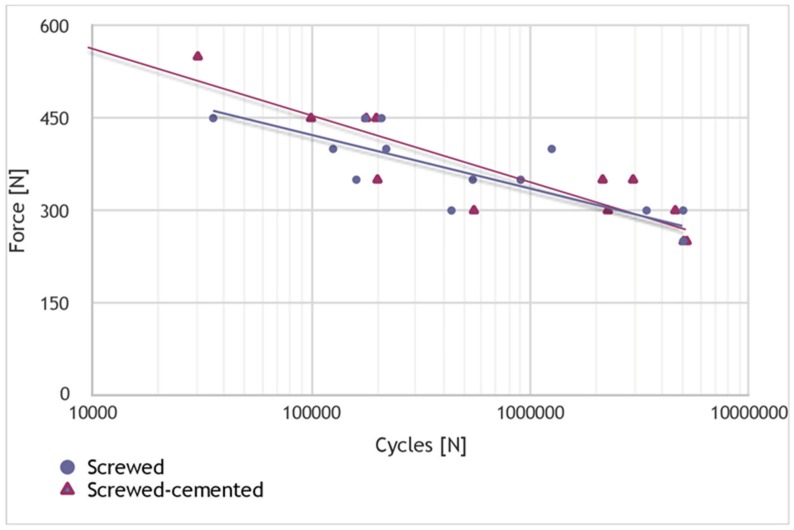
S-N curve. Chart showing the applied load as a function of the number of cycles. After the samples underwent five million cycles, the test was stopped. The horizontal and vertical lines are used as a guide for the eye.

The stress-number of cycles (S-N) curve results, as well as the failure modes, are presented in Table 2 and Table 3 for group A (screwed restoration) and group B (screwed-cemented restoration), respectively. In both groups, when the applied load was equal to 250 N, the three specimens tested (samples 13–15) remained intact after five million cycles (Table 2 and Table 3). The S-N curves for both groups are presented in Figure 1. However, despite the small number of samples, an unequivocally similar trend of both specimen groups on the S-N plot, could be noted.

**Figure 2:**
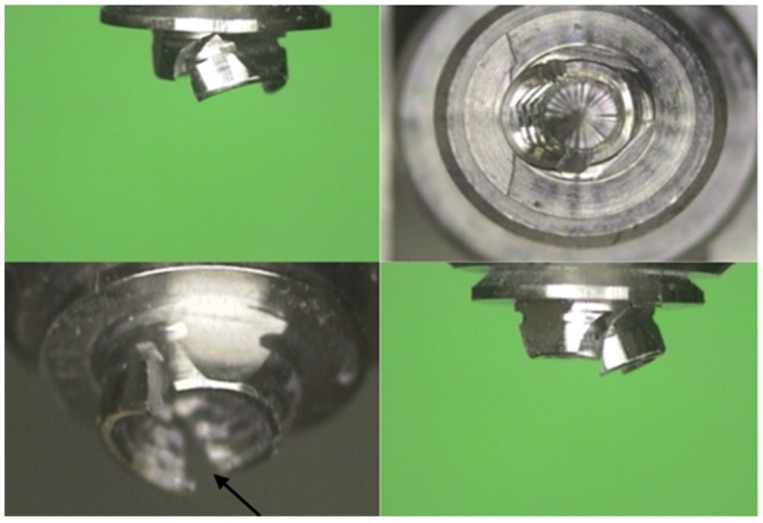
Specimen number seven in group A shows deformation and fracturing within the cone of the multi-unit abutment (MUA) under an applied load of 350 N. This mode of failure was predominant in group A.

**Figure 3:**
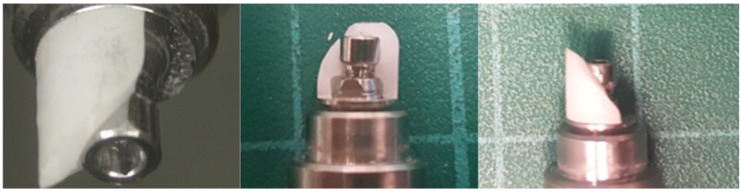
Specimen number one in group A shows fracturing of the zirconia concomitantly with bending of the restoration screw head under an applied load of 450 N. This failure was observed in group A at only high loading forces.

**Figure 4:**
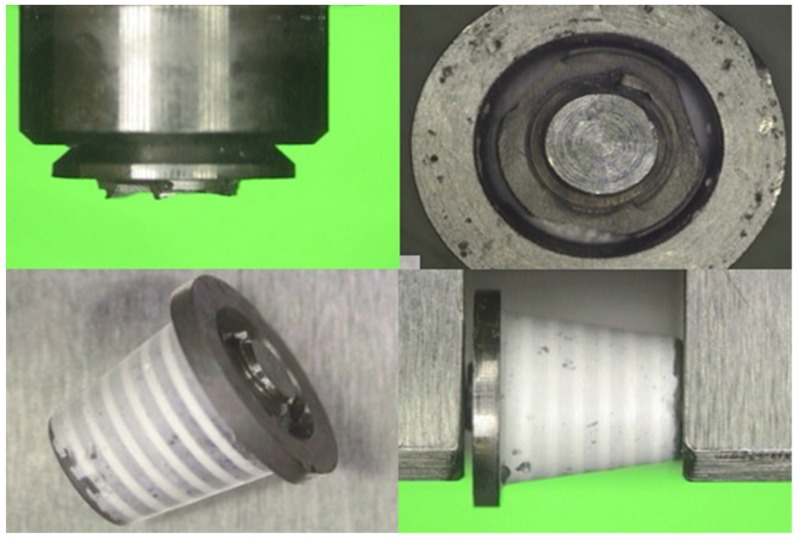
Specimen number 10 in group B exhibits adhesive failure between the zirconia and T-base in addition to deformation and fracturing within the cone of the MUA under an applied load of 300 N. This mode of failure was predominant in group B.

Stereomicroscopic examination of the failure mode of all the specimens revealed differences between the groups (Table 2 and Table 3). Group A exhibited mainly failure within the metal (MUA/screw), and zirconia failure occurred only at a high loading force. The representative failure photographs are presented in Figure 2 and Figure 3. Figure 2 shows deformation and fracturing within the cone of the MUA, and Figure 3 shows fracturing of the zirconia concomitantly with bending of the restoration screw head. Group B exhibited failure within the metal (the cone of the MUA/the restoration screw head), mostly in conjunction with adhesive failure between the zirconia and T-base. Figure 4 shows adhesive failure between the zirconia and T-base in addition to fracturing within the MUA cone, which was the predominant failure mode in group B.

## 3. Discussion

Numerous studies have evaluated the fracture strength of different abutments for cemented implant restorations. No available data exists on the fracture strength of screwed implant restorations connected to MUAs, even though these types of restorations have gained popularity with the increased use of immediate loading of multiple implants.

The current study compared the static and dynamic load-bearing capacities and determined the failure mode of zirconia crowns screwed to MUAs with and without a T-base, an issue that has not yet been addressed in the literature. Our null hypothesis was partially proven because both groups demonstrated that three specimens survived 5,000,000 cycles, according to International Standard (ISO) 14801, at 250 N. This result may indicate that the two modes of retention (with and without a T-base) have no effect on the mechanical performance of the prosthesis under cyclic loading.

The range of human biting forces is 50–300 N in normal chewing as opposed to 175–800 N for maximum voluntary biting forces [19,20,21]. These values are highly dependent on the measurement tools, mainly strain gauges, piezo-electric sensors, and pressure sheets. In the current study, the occlusal forces range from 250 N to 550 N, but the specimens survived 5,000,000 cycles only at 250 N. Indeed, the 250 N value fits well within the range of normal chewing forces but should be interpreted with caution because this value is valid for the specific configuration of the current study design. The conclusion that zirconia screw-type prostheses, with and without a titanium base, are equally adequate for implant-supported prostheses with MUAs must be based on the unequivocally similar trend in the S-N plots of both specimen groups and not on the 250 N value.

On the other hand, the failure modes differ between the groups. Metal failure (MUA/screw) was demonstrated in both groups; however, in group A (screwed), zirconia failure was also observed, mainly at high loading forces. This pattern was not observed in group B (screwed-cemented). With the latter failure pattern, which included metal failure, most specimens exhibited an adhesive mode of failure between the cement and zirconia. In this study, we used 3Y-TZP, although a newer form of zirconia material with better translucency (5Y-TZP) has been introduced. Research has shown that 3Y-TZP has significantly higher flexural strength than that of 5Y-TZP [22]. Our purpose in the current study was to compare both restorations for posterior clinical use; in that case, 3Y-TZP was preferred.

This research was performed according to ISO standard 14801 because the international standard requires that stress is exerted on the implant-abutment-restoration complex in the “worst-case” scenario loading situation [23]. In addition, the ISO standard established a methodology that can be replicated by other researchers studying the same subject. Studies applying dynamic loading tests do not always comply with ISO 14801. Koyama et al. [24] and Ding et al. [25] used the “staircase method” to determine the mean cyclic fatigue force in accordance with the current study. However, Duan et al. [26] used variable loading amplitude, and each specimen was subjected to increasing load amplitude over time and thus experienced multiple load amplitudes. Shemtov-Yona and Rittel [27] suggested a new method for the dynamic loading test based on random spectrum loading, noting that the ISO 14801 protocol is questionable for statistical analysis.

In the screwed-cemented implant-supported restoration, the impact of oral fluids on the solubility of the luting cement and the retention of the restoration is always of concern. To simulate the oral cavity environment, artificial aging, such as water storage and thermocycling, is performed. In the current study, an aging protocol of storage at 37 °C under 100% humidity for one month, followed by thermal cycling, was conducted. Naumova et al. [28] and Güngör and Nemli [29] investigated the effect of various resin cements on the retentive strength of zirconia abutments bonded to titanium inserts (T-bases). Both studies showed that exposure to the liquid environment had a negative effect on the retention force of the resin cements, regardless of whether thermocycling was performed. The specimens were sandblasted, after which a primer was applied to some of the specimens. Irrespective of the surface treatment, the typical mode of failure was adhesive failure. In contrast, Zenthofer et al. [30] demonstrated that artificial aging had no effect on the retentive force of the resin cement, which might be influenced by the type of luting cement. In the current study, we used a self-curing resin-based cement (Multilink Hybrid Abutment cement, Ivoclar Vivadent). Güngör and Nemli [29] investigated the effect of three types of resin cement on the retentive strength of zirconia abutments bonded to titanium inserts and found that the retentive force of the multilink hybrid abutment cement was the same as that of Panavia F 2.0 but was lower than that of the Zirconite cement.

The failure mode in group B (screwed-cemented restoration, with a T-base) was expected to be mainly adhesive failure. However, our results demonstrated that at all force levels, metal failure also occurred (screw/MUA). This result is in agreement with the results of other reports [31,32,33], which differ from our study because they evaluated the strength of the zirconia abutments with and without a T-base, while we evaluated the zirconia prostheses with and without a T-base. Metal failure was also observed at all force levels in group A (without a T-base). One would expect that because there was no luting cement, zirconia failure would occur in all the specimens, as this was the rationale for using the T-base. Our results showed that zirconia failure occurred at only high loading forces. This phenomenon is probably related to the high flexural strength (900–1200 Mpa) and fracture toughness (9–10 Mpa m^0.5^) of the Y-TZP monolithic structure [3]. Under continuous cyclic loading, a catastrophic failure will occur within the metal (titanium) at lower force levels than those affecting the zirconia. At higher forces, because zirconia is a brittle material that is not capable of deforming, catastrophic failure will occur within the zirconia concomitantly with metal failure. When there is a cement mediator, as in group B, the weakest link is the cement, and thus an adhesive failure will occur and cause the zirconia to separate from the titanium sleeve, even at high force levels. Zirconia failure was thus not observed.

From a clinical point of view, separation of the zirconia crown from the T-base, as was found in the screwed-cemented restoration, is easy to repair by re-cementation. Even, metal failure, either of the MUA or of the screw head, is reparable with some cost, by simply replacing the damaged elements. However, when a zirconia fracture occurs, as was seen in the screwed restoration (without a T-base) at high loading forces, the zirconia crown should be replaced, with a higher cost.

The limitation of this study was the number of experiments, which was too small for full-scale statistical analysis. However, this study highlighted an importance issue that has not yet been addressed in the literature. Because this is a pilot study, further studies with larger sample sizes are warranted. In addition, similar to other in vitro studies, a laboratory study design cannot fully reproduce clinical conditions, even if the samples are artificially aged by chewing simulation and thermocycling. One might argue that the geometry of the hemisphere with the zirconia coping is different from the geometry of the in vivo zirconia crown. We followed the ISO protocol that simulates the functional loading of an endosseous dental implant and its prosthesis under “worst-case” conditions; however, this method cannot fully predict the in vivo behavior of the dental implant and its prosthesis. Therefore, the results of the present study should be interpreted with caution.

## 4. Materials and Methods

**Figure 5:**
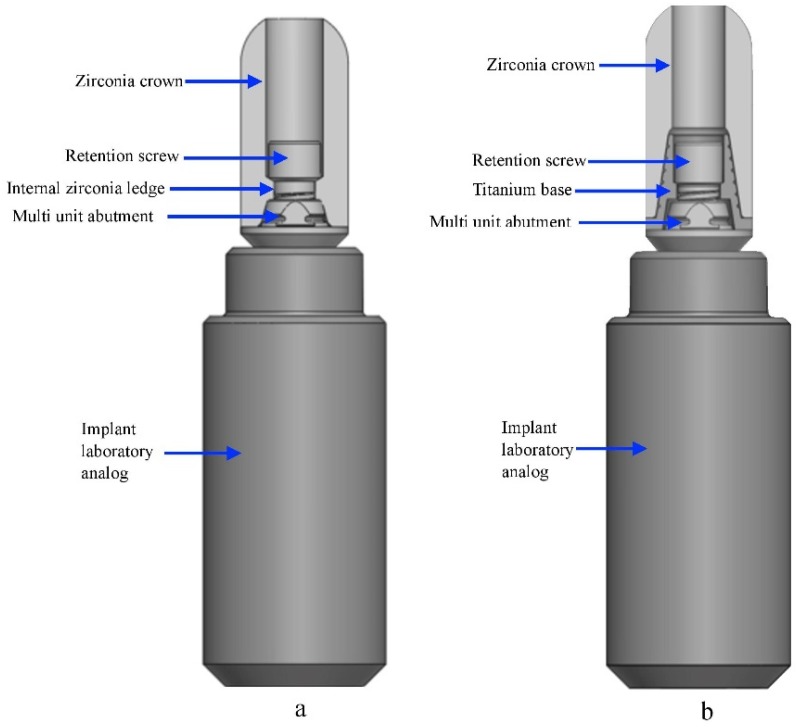

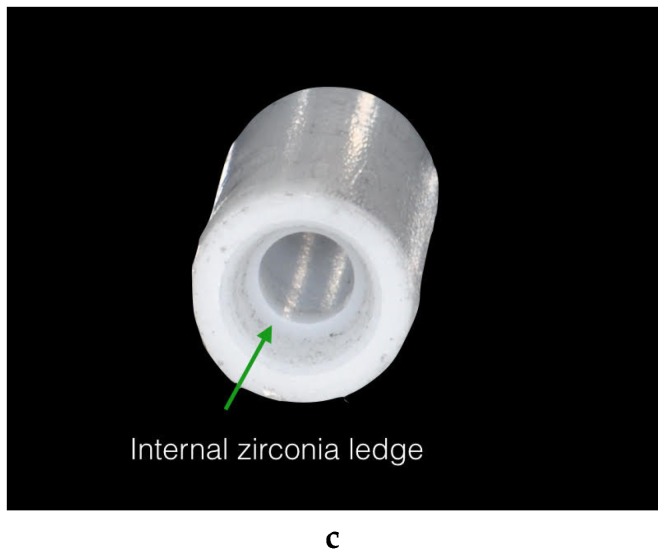
Components of the zirconia-abutment restorations. (**a**) Screwed restoration; (**b**) screwed-cemented restoration; (**c**) apical view of the screwed zirconia cap. Note the internal zirconia ledge for the support of the screw head.

Thirty-six straight MUAs (Alpha-Bio Tec., Petah Tikva, Israel) were torqued to laboratory analogs (6 mm width) (Alpha-Bio Tec.) with a digital torque ratchet (30 Ncm) (CEDAR Model DIW 15) and were assigned to two groups (24 specimens per group):

Group A (screwed restoration) (n = 18): Spherically shaped monolithic zirconia copings (Ceramill^®^ material, Amann Girrbach AG, Koblach, Austria) with a height of 10 mm and a wall thickness of 1.5 mm were designed and milled with an internal ledge for the screw head and then torqued to the MUAs with a digital torque ratchet (25 Ncm) (CEDAR Model DIW 15) (Figure 5a).

The screwed zirconia cap has an internal zirconia ledge that supports the screw head and is in intimate contact with the MUAs (Figure 5c).

Group B (screwed-cemented restoration) (n = 18): Spherically shaped monolithic zirconia copings (Ceramill^®^ material, Amann Girrbach AG, Koblach, Austria) with a height of 10 mm and a wall thickness of 1.5 mm were designed and milled to fit a titanium sleeve (a T-base) (Alpha-Bio Tec.) (Figure 5b). The cementation of the monolithic zirconia crowns to the T-base was performed according to commonly accepted protocols [34]. The intaglio surface of the crowns and the outer surface of the T-base were airborne particle-abraded using 50 µ alumina oxide particles at pressures of 1 and 2 bar, respectively; then, the surfaces were cleaned with alcohol. The pretreated T-base and the intaglio surface of the crown were coated with a single component primer to promote adhesion (Monobond Plus, Ivoclar Vivadent). The primer agent was allowed to react for 60 min, after which it was dispersed by a stream of air. The specimens were then cemented to the T-base with a self-curing resin-based cement (Multilink Hybrid Abutment cement, Ivoclar Vivadent) according to the manufacturer’s instructions. The copings were seated with a device that allowed a predefined pressure of 50 N to be applied along the longitudinal axis of the abutment for 7 min [35,36]. Excess resin was removed from the bonded margins before it set completely. After cementation, the specimens were torqued to the MUAs with a digital torque ratchet (25 Ncm) (CEDAR Model DIW 15).

All specimens in both groups were stored at 37 °C under 100% humidity for one month, followed by thermal cycling between water temperatures of 5 °C and 55 °C for 20,000 cycles with a 10 s dwell time (Y. Manes, Tel-Aviv, Israel).

**Figure 6:**
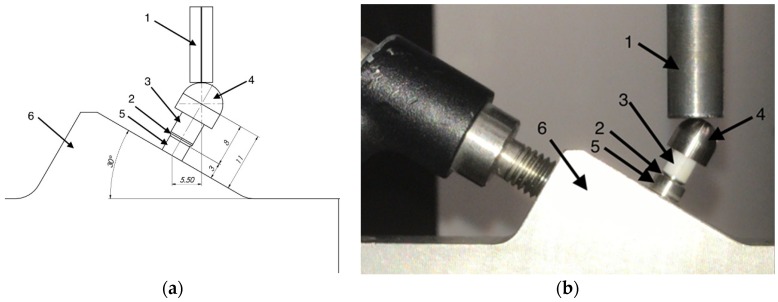
Test setup following ISO 14801:2016. (**a**) Schematic illustration of the test design: (1) loading device; (2) nominal bone level; (3) zirconia coping; (4) hemispherical loading member; (5) dental implant analog; and (6) metal specimen holder; (**b**) setup for the study.

After aging, a fatigue test was performed according to the ISO standard 14801 [23]. To comply with this standard, every specimen was embedded in a designated metal holder inclined 30° to the longitudinal force axis direction. Additionally, to allow a uniform stress distribution and to avoid stress concentration, the load was applied to a hemisphere modeled exactly on the occlusal surface of each coping without cementation (Figure 6a,b).

To determine the static maximum load that the assembly could resist, which corresponds to one cycle on the S-N curve (plotted as force versus log number of cycles), three samples of each group were subjected to an increased static compressive load at a crosshead speed of 1 mm/min until catastrophic failure using a uniaxial universal testing machine (Instron, ElectroPuls E3000, Instron Corp., Buckinghamshire, high Wycombe, UK). The average failure rate from this test was calculated and defined as the maximal failure load.

For the dynamic loading, the remaining specimens were tested in progressively lower loads, starting at 80% of the maximal failure load, using an MTS Systems Corporation machine (Instron, ElectroPuls E3000, Instron Corp., Buckinghamshire, high Wycombe, UK) (Table 2 and Table 3). The compressive fatigue limits were determined by testing according to the staircase or up-and-down method [37] in a 14 Hz frequency cycle. In this method, the tests were conducted sequentially, with the maximum applied stress in each successive test increasing or decreasing by a fixed amount according to whether the previous stress resulted in failure, until a load was reached at which three specimens survived 5,000,000 cycles, which is equivalent to five years of in vivo mastication [38]. The failure mode of all the specimens was evaluated by stereomicroscopy at 20× magnification (StarLite 150, OGP, Rochester, NY, USA).

Due to the small number of samples, no statistical analysis could be performed with sufficient statistical power.

## 5. Conclusions

Within the limitations of this pilot study, zirconia restoration screwed directly to an MUA without a T-base is shown to be a viable procedure, and the survival odds of this restoration are not inferior to those of the screwed-cemented restoration. Failure will probably occur within the metal (MUA/screw) before zirconia failure. Further studies with larger sample sizes are warranted.

## Figures and Tables

**Table 1:** Mean maximal load-bearing capacity (N) and 80% level for each experimental group.

Specimen	Group A(N)	Group B(N)
1	872	**718**
2	811	**680**
3	675	**707**
Mean	786	**702**
80% Level	629	**561**

Group A—screwed restoration; Group B—screwed-cemented restoration; 80% Level—80% of the average static load compressive test result.

**Table 2:** Fatigue test results for group A (screwed restoration).

Specimen	Load (N)	No. of Cycles	Average No of Cycles	Failure Mode
1	450	35,670	-	Zirconia + metal (screw)
2	450	209,543	80,545	Metal (MUA + screw) Zirconia + metal (screw)
3	450	176,422	-	
4	400	1,254,436	-	Metal (MUA + screw)
5	400	125,878	533,457	Metal (MUA + screw)
6	400	220,056	-	Metal (MUA + screw)
7	350	160,749	-	Metal (MUA + screw)
8	350	546,871	266,048	Metal (MUA + screw)
9	350	905,205	-	Metal (MUA + screw)
10	300	3,401,784	-	Metal (MUA + screw)
11	300	437,068	2,946,284	Metal (MUA + screw)
12	300	5,000,000	-	Metal (MUA + screw)
13	250	5,000,000	-	No Failure
14	250	5,000,000	5,000,000	No Failure
15	250	5,000,000	-	No Failure

With a maximum load of 250 N, three specimens completed five million cycles without fracturing; Load—maximum load applied in each cycle; Number of cycles—number of cycles before the test was interrupted; Average number of cycles- for particular load values; MUA—multi-unit abutment.

**Table 3:** Fatigue test results for group B (screwed-cemented restoration).

Specimen	Load (N)	No. of Cycles	Average No of Cycles	Failure Mode
1	550	6607	-	Adhesive + metal (screw)
2	550	30,341	22,450	Metal (screw) Adhesive + metal (screw)
3	550	30,341	-	
4	450	99,751	-	Adhesive + metal (MUA)
5	450	178,367	158,818	Metal (screw)
6	450	198,337	-	Adhesive + metal (MUA)
7	350	2,950,685	-	Adhesive + metal (screw)
8	350	200,751	1,764,689	Adhesive + metal (MUA)
9	350	2,142,632	-	Adhesive + metal (MUA)
10	300	553,418	-	Adhesive + metal (MUA)
11	300	2,264,147	2,472,765	Metal (screw)
12	300	4,600,732	-	Adhesive + metal (MUA
13	250	5,000,000	-	No Failure
14	250	5,000,000	5,000,000	No Failure
15	250	5,000,000	-	No Failure

With a maximum load of 250 N, three specimens completed five million cycles without fracturing; Load—maximum load applied in each cycle; Number of cycles—number of cycles before the test was interrupted; Average number of cycles- for particular load values; MUA—multi-unit abutment.
